# Preliminary validation of an Italian version of the Athlete Burnout Questionnaire

**DOI:** 10.3389/fpsyg.2026.1758214

**Published:** 2026-03-20

**Authors:** Alice Valdesalici, Erika Borella, Andrea Spoto, Marta Ghisi

**Affiliations:** 1 Department of General Psychology, University of Padua, Padua, Italy; 2 U.O.C. Hospital Psychology, University-Hospital of Padua, Padua, Italy

**Keywords:** athlete burnout, factorial validity, psychometrics, questionnaire validation, sport

## Abstract

**Introduction:**

Burnout in athletes is a growing concern in sport environments. There is a need for a psychometrically sound assessment tool to examine this phenomenon in Italian sport contexts. The purpose of the present study was to examine the psychometric properties of an Italian version of the Athlete Burnout Questionnaire.

**Methods:**

A sample of 255 athletes (M = 23.7, SD = 4.1; 32.9% women) of varied sports completed an Italian version of the ABQ along with measures assessing distress and resilience. The factor structure of the test was inspected by comparing the fit of three confirmatory factor analysis models. Internal consistency of the questionnaire was inspected, as was its construct validity.

**Results:**

The confirmatory factor analysis showed that the first-order model with three correlated factors presented an acceptable fit to the data and a better fit compared to the alternative models. The results also showed scores on the respective burnout dimensions to have good internal consistency. Furthermore, the pattern of relationships of the ABQ subscales with the measures of distress and resilience supported the construct validity of the Italian version of the ABQ. As expected, the three dimensions of burnout were significantly positively correlated with distress and significantly negatively correlated (or non-correlated) with resilience.

**Conclusion:**

These findings support the three-factor multidimensional model of burnout, offering preliminary evidence for the reliability and validity of an Italian version of the ABQ.

## 1 Introduction

Burnout has become a salient issue in competitive sports, where increasing performance expectations and intense training regimens expose athletes to chronic physical and psychological demands. Within the sport psychology literature, athlete burnout is widely recognized as a multidimensional syndrome encompassing emotional and physical exhaustion, a reduced sense of athletic accomplishment, and devaluation of sport participation (Raedeke and Smith, 2001). These dimensions reflect a progressive erosion of the athlete’s passion and commitment, with potentially detrimental effects on athletic performance, mental health, and long-term engagement in sport (Glandorf et al., 2023).

The origins of the burnout construct can be traced back to Freudenberger’s (1974) description of the emotional depletion experienced by healthcare professionals. Maslach et al. (1996) later conceptualized burnout as a syndrome composed of emotional exhaustion, depersonalization, and reduced personal accomplishment, primarily within occupational settings. Over time, this framework was extended beyond the helping professions to include students, caregivers, and eventually athletes, as researchers recognized similar patterns of chronic stress and motivational decline (Maslach et al., 2001; Schaufeli and Enzmann, 1998). Approximately in the same period, Raedeke (1997) proposed a definition of burnout specific to the sport context to reflect the central role performance has for athletes. According to Raedeke, athlete burnout is characterized by: (i) emotional and physical exhaustion, stemming from the chronic physical and psychological demands of training and competition; (ii) a reduced sense of accomplishment, reflecting feelings of inefficacy and perceived failure to meet performance standards; and (iii) sport devaluation, marked by a negative and detached attitude toward sport participation (Raedeke, 1997; Raedeke and Smith, 2001). Aligned with this conceptualization, Raedeke and Smith (2001, 2009) developed the *Athlete Burnout Questionnaire (ABQ),* a self-report instrument designed to measure burnout perceptions in sports. The ABQ asks athletes “How often do you feel this way?” and presents 15 items that measure perceptions of exhaustion (e.g., “I am exhausted by the mental and physical demands of sport”), reduced athletic accomplishment (e.g., “It seems no matter what I do, I don’t perform as well as I should”), and devaluation of the sport context (e.g., “I don’t care as much about my sport performance as I used to”). Each dimension is composed of five items that are rated on a 5-point Likert scale ranging from “almost never” to “almost always.” Since its development, the ABQ has become the most widely used instrument for measuring athlete burnout and has been validated across several languages, including German, Spanish, French, and Chinese (Arce et al., 2012; Gerber et al., 2018; Isoard-Gautheur et al., 2010; Liu et al., 2022), among others. Across these studies, the three-factor structure originally proposed by Raedeke and Smith (2001) has been consistently replicated (Guedes and de Souza, 2016; Martínez-Alvarado et al., 2019; Raedeke and Smith, 2009). Some studies have also explored alternative factor structures, such as bifactor and higher-order models, and employed alternative techniques, including exploratory structural equation modeling (ESEM), to further examine the instrument’s psychometric properties (Grugan et al., 2024; Isoard-Gautheur et al., 2010; Liu et al., 2022). In addition, the ABQ has demonstrated strong internal consistency, construct validity, test–retest reliability, and measurement invariance (Cresswell and Eklund, 2006; Grugan et al., 2024; Raedeke and Smith, 2009).

Understanding how burnout develops in athletes is particularly important given the unique psychological, social, and organizational demands of the sport environment. Many athletes begin intensive training at a young age, undergo early specialization, and experience substantial emotional and financial investment from coaches, families, and sport systems (Gould and Whitley, 2009; Gustafsson et al., 2011). These pressures can contribute to chronic stress and a sense of entrapment - core elements in the development of burnout (Gustafsson et al., 2011). Empirical research has linked athlete burnout to a range of individual and contextual risk factors, including dysfunctional perfectionism (Madigan et al., 2015), maladaptive motivation (Gustafsson et al., 2018; Lemyre et al., 2008), and low satisfaction of basic psychological needs such as competence, autonomy, and relatedness (Shannon et al., 2022; Woods et al., 2022). Environmental stressors such as excessive training loads, interpersonal conflicts with coaches or teammates, and insufficient recovery time have also been shown to play a role (Goodger et al., 2007; Pacewicz et al., 2019). In addition, psychological traits like perfectionistic concerns, chronic perceived general distress, negative affectivity, and anxiety are positively associated with burnout symptoms (Dišlere et al., 2025; Woods et al., 2022). Conversely, protective factors include adaptive coping strategies, perceived social support, and resilience-related skills, all of which can buffer athletes from burnout (Dišlere et al., 2025; Pacewicz et al., 2019; Yu et al., 2025).

In the Italian context, research on athlete burnout has recently gained traction, particularly in relation to youth sports. Studies have explored the relationship between burnout and variables such as motivational climate, resilience, and psychobiosocial experiences (Morano et al., 2022; Vitali et al., 2015). Despite the growing interest within the Italian sport context, a standardized and psychometrically validated Italian version of the ABQ has not yet been published. As a result, researchers have often relied on translated versions without formal validation, which may affect the reliability and comparability of findings. Furthermore, existing implementations of these Italian translations have predominantly concentrated on youth athletes, highlighting the need for a validated instrument that is also appropriate for adult populations. A psychometrically sound Italian version of the ABQ would provide a reliable tool for both researchers and practitioners to assess burnout symptoms in Italian-speaking athletes, monitor changes over time, and evaluate the effectiveness of interventions aimed at reducing burnout risk.

Thus, the aim of the current study was to translate and preliminarily validate an Italian version of the ABQ. The first goal was to test the factorial structure of the Italian ABQ through confirmatory factor analysis. Consistent with the original conceptualization, we first tested the three-factor structure and subsequently evaluated alternative specifications, namely a unidimensional model and a bifactor model, as suggested in previous validation studies (e.g., Liu et al., 2022). Bifactor models have been increasingly applied in psychometric research to assess the multidimensionality of constructs and the coexistence of a general overarching factor with domain-specific dimensions (Reise, 2012). A second goal was to evaluate the questionnaire’s reliability and construct validity by examining the relationship with theoretically related constructs, specifically general distress and resilience. This work represents an initial step toward improving the assessment and understanding of burnout in Italian-speaking athletes and enhancing mental health support in the sport domain.

## 2 Methods

### 2.1 Participants

A total of 256 athletes participated in the survey. One athlete did not complete the ABQ and was therefore excluded from the present analyses, resulting in a final sample of 255 participants. The final sample (*N* = 255; 171 males and 84 females) included athletes competing in various sports (e.g., rugby, soccer, track & field, basketball, volleyball), aged 18–39 years (M = 23.7, SD = 4.1). Among them, 110 reported being both athletes and students, 51 were athletes who also worked, and 34 reported both studying and working in addition to their athletic careers. Most of the athletes participated in team sports (*N* = 182, 72.5%), while the rest participated in individual sports. Participants were active athletes competing at different levels. Most of them competed at a national (*N* = 159, 62.4%) or international level (*N* = 73, 28.6%), while the minority competed at lower levels (*N* = 23, 9.0%). Athletes reported an average of 13.7 years (SD = 4.9) of involvement in their sport.

### 2.2 Translation of the athlete burnout questionnaire

The ABQ was translated into Italian using the standard forward–backward translation procedure (Brislin, 1986). Firstly, three researchers independently translated the original English version of the ABQ into Italian. A synthesis of the translations was later reviewed by an expert in sport psychology to reach a common version. After this, a separate bilingual translator with extensive knowledge of psychological research, blind to the original version, back-translated the shared version into English. The back-translation proved to be nearly identical to the original one.

### 2.3 Measures

Participants completed sociodemographic questions (e.g., age, sex, gender, years of education, marital status, occupation) and questions about their involvement in sports (e.g., discipline, years of practice, competitive level). Participants were also asked to complete a series of self-report questionnaires investigating athlete burnout, general distress, and resilience.

#### 2.3.1 Athlete burnout

The ABQ (Raedeke and Smith, 2001) is a 15-item scale assessing the three components of athlete burnout: physical and emotional exhaustion (PEE – five items), reduced sense of accomplishment (RSA – five items), and sport devaluation (DEV – five items). Responses are rated on a 5-point Likert scale ranging from “almost never” (1) to “almost always” (5). Items 1 and 14 are reverse-scored.

#### 2.3.2 General distress

The Italian version of the Depression Anxiety Stress Scales-21 (DASS-21) (Bottesi et al., 2015; Lovibond and Lovibond, 1995) is a 21-item measure assessing anxious, stress, and depressive symptoms over the past week. Responses are rated on a 4-point Likert scale ranging from “did not apply to me at all” (0) to “applied to me very much” (3). Examples of items include “I found it difficult to relax,” “I felt I was close to panic,” and “I felt that life was meaningless”. In the current study, the composite score was used, which reflects the level of perceived general distress. Internal consistency of the composite score was satisfactory in previous research (*α* = 0.90; Bottesi et al., 2015). In the present sample, the composite score showed good internal consistency (α = 0.90, *ω* = 0.92).

#### 2.3.3 Resilience

The Italian version of the Connor-Davidson Resilience Scale-10 (CD-RISC-10) (Campbell-Sills and Stein, 2007; Di Fabio and Palazzeschi, 2012) is a 10-item scale measuring the ability to bounce back from adversity. Responses are rated on a 5-point Likert scale ranging from “not true at all” (0) to “true nearly all the time” (4). Example items include being “able to adapt to change” or “tend to bounce back after illness or hardship”. A total score is computed that reflects the level of resilience. Internal consistency of the scale was satisfactory in previous research (α = 0.89; Di Fabio and Palazzeschi, 2012). In the present sample, the total score showed good internal consistency (α = 0.85, ω = 0.88).

### 2.4 Procedure

Athletes were recruited through word of mouth and an academic office that distributed the study invitation to student-athletes via its internal mailing list. Participants who voluntarily decided to take part in the study were provided with a link to access an online survey, which included an online informed consent outlining the study’s objectives and the participants’ right to withdraw at any point without penalty. After reading and accepting the informed consent, participants were invited to complete the questionnaires autonomously and independently through the online survey. At the beginning of the survey, clear instructions were given to complete the survey. No compensation was offered for participation. Inclusion criteria included being an Italian speaker, aged 18 or older, and currently engaged in a competitive sport.

The present study was conducted in accordance with the Declaration of Helsinki and ethics approval was obtained from the relevant departmental ethics committee at the local School of Psychology.

### 2.5 Data analysis

Data were analyzed using R (version 4.4.0; R Core Team, 2024). As a preliminary step, means, standard deviations, skewness, and kurtosis were calculated for all ABQ items to assess data distribution. Differences in observed ABQ mean scores were also explored across gender, sport type, and competitive levels.

A sample of 255 participants was considered adequate to test the factorial structure of a 15-item instrument (5 indicators per factor), meeting commonly cited minimum recommendations for confirmatory factor analysis (CFA) (i.e., approximately 200 cases or 10 participants per item; Everitt, 1975; Kline, 2016). Simulation studies suggest that samples in the range of 200–300 generally provide adequate parameter stability and power for CFA models of moderate complexity (Wolf et al., 2013), and RMSEA-based sensitivity analyses indicate sufficient power to distinguish close from poor model fit in models with comparable degrees of freedom (MacCallum et al., 1996).

Consequently, to examine the factorial validity of the Italian version of the ABQ, three CFA models were tested and compared using the R package lavaan (Rosseel, 2012): (1) a first-order model with a single global burnout factor; (2) a first-order model with three correlated factors representing the dimensions of burnout (physical/emotional exhaustion, reduced sense of accomplishment, and sport devaluation); and (3) a bifactor model including one general burnout factor and three orthogonal factors. A second-order model (i.e., three dimensions plus a common higher-order burnout factor) was not tested, as this would have produced an identical fit as the three-factor oblique model (Brown, 2015). In the CFA, factor variances were fixed to 1.0 to allow model identification and obtain standardized estimates of factor loadings. Given the distribution of the data, the diagonally weighted least squares (DWLS) estimator was employed in all CFAs. Model fit was evaluated using standard fit indices: the Chi-square value, Comparative Fit Index (CFI), Tucker-Lewis Index (TLI), Root Mean Square Error of Approximation (RMSEA), and Standardized Root Mean Square Residual (SRMR). Specifically, a threshold of >0.95 for the CFI and TLI, of close to (or lower than) 0.08 for the SRMR and RMSEA represented an adequate fit (Hu and Bentler, 1999).

Internal consistency of the subscales was assessed by computing ordinal Cronbach’s alpha and McDonald’s omega coefficients. Lastly, construct validity was evaluated by computing Spearman’s correlations between the ABQ subscales and scores of general distress and resilience.

## 3 Results

### 3.1 Descriptive analyses

As a preliminary step, descriptive statistics and item distributions for the Italian version of the ABQ were examined. The Shapiro–Wilk test indicated that none of the items followed a normal distribution. Additionally, Mardia’s test revealed a significant departure from multivariate normality. Descriptive statistics are reported in Table 1.

**Table 1:** Descriptive statistics of the Italian translated Athlete Burnout Questionnaire (*n* = 255).

Factor	Item	Mean (SD)	Skewness	Kurtosis
Physical/emotional exhaustion	2	2.85 (0.93)	−0.18	−0.06
	4	2.59 (1.00)	0.10	−0.57
	8	1.96 (0.99)	0.68	−0.44
	10	2.14 (1.12)	0.74	−0.25
	12	2.16 (1.04)	0.58	−0.36
Reduced sense of accomplishment	1	2.45 (0.79)	0.43	0.39
	5	2.22 (0.95)	0.26	−0.68
	7	2.75 (1.03)	0.00	−0.53
	13	2.57 (1.11)	0.21	−0.63
	14	2.80 (0.91)	0.31	0.22
Sport devaluation	3	1.84 (0.94)	0.93	0.29
	6	1.86 (1.12)	1.11	0.24
	9	1.79 (1.03)	1.03	−0.11
	11	2.51 (1.26)	0.25	−1.08
	15	1.65 (0.87)	1.03	−0.11

SD, standard deviation.

Differences in observed mean scores on the ABQ dimensions across key sample subgroups (i.e., gender, sport type, and competitive level) were also explored. Significant differences in PEE emerged both for gender (t (253) = −2.01, *p* = 0.045, Cohen’s d = −0.27) and sport type (t (253) = −2.31, *p* = 0.021, Cohen’s d = −0.32), with male athletes and team sports athletes reporting slightly higher scores. No significant differences were observed for RSA and DEV with respect to gender and sport type (all *p* > 0.05). Moreover, significant effects of competitive level were found for RSA (*F* (2, 252) = 8.07, *p* < 0.001, η^2^p = 0.06) and DEV (F (2, 252) = 5.47, *p* = 0.005, η^2^p = 0.04), but not for PEE. Post-hoc analyses indicated lower RSA scores among international-level athletes compared to athletes competing at both national and regional levels, and lower DEV scores compared to national-level athletes.

### 3.2 Factor structure

To identify the factorial structure of the Italian ABQ, three different CFA models were tested. Table 2 reports fit indices for each tested model.

**Table 2:** Model fit indices of measurement models.

Model	χ2 (df)	CFI	TLI	RMSEA	SRMR
M1	1086.204 (90)	0.876	0.855	0.210	0.160
M2	366.756 (87)	0.965	0.958	0.113	0.099
M2 (modified)	221.448 (85)	0.983	0.979	0.080	0.082
M3	166.787 (75)	0.989	0.984	0.070	0.071

M1, one-factor model; M2, three-correlated-factors model; M2 (modified), three-correlated-factors model with correlated residuals between item 2 and 4 and between item 1 and 14; M3, bi-factor model; CFI, comparative fit index; TLI, Tucker-Lewis index; RMSEA, root mean square error of approximation; SRMR, standardized root mean square residual.

**Figure 1:**
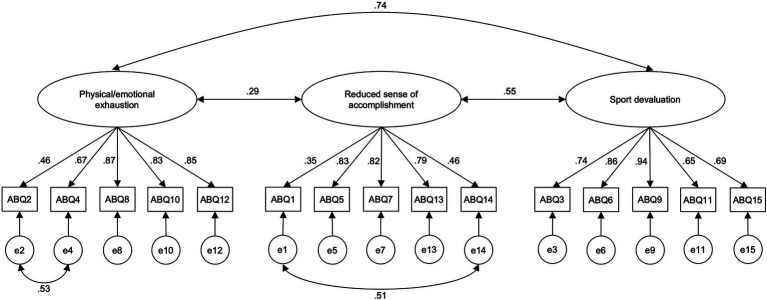
Confirmatory factor analysis (CFA) model of an Italian version of the Athlete Burnout Questionnaire (ABQ) showing the modified three-factor structure. Curved arrows between latent variables represent factor correlations, curved arrows between selected error terms indicate correlated residuals.

The unidimensional model showed the worst fit to the data (χ2 (90) = 1085.649, *p* < 0.001, CFI = 0.880, TLI = 0.860, RMSEA = 0.209, SRMR = 0.159), compared to the other two models. The three-factor model showed a better but still suboptimal fit (χ2 (87) = 373.464, *p* < 0.001, CFI = 0.965, TLI = 0.958, RMSEA = 0.114, SRMR = 0.099), with some indices exceeding conventional thresholds. An inspection of the standardized residuals and modification indices revealed local misfit in the model. In particular, items 2 and 4 (PEE dimension) and items 1 and 14 (RSA dimension) appeared to share variance not accounted for by the model, likely due to their overlapping content. Based on both statistical indications and theoretical considerations, the model was re-specified by sequentially allowing correlated residuals between these item pairs. The modified three-factor model showed improved global fit (χ2 (85) = 229.333, *p* < 0.001, CFI = 0.983, TLI = 0.978, RMSEA = 0.082, SRMR = 0.083), with most fit indices meeting cutoff criteria. As shown in Table 3, all factor loadings were significant at *p* < 0.05 and ranged from 0.35 to 0.94. Inter-factor correlations revealed strong associations between DEV and the other factors (PEE: r = 0.74; RSA: r = 0.55), while the correlation between PEE and RSA was relatively weak (r = 0.29). The bifactor model exhibited the best overall fit (χ2 (75) = 169.782, *p* < 0.001, CFI = 0.989, TLI = 0.984, RMSEA = 0.071, SRMR = 0.071). However, this model also revealed some negative or very low loadings (e.g., items 3 and 15) on either the specific dimensions or the general factor. It is worth noting that bifactor models are known to often overfit the data, potentially inflating fit indices without necessarily reflecting a theoretically meaningful structure. The modified three-factor model (Figure 1), instead, showed adequate fit and consistent, significant loadings, supporting both theoretical coherence and psychometric adequacy. Considering all, the modified three-factor model was retained as the factor structure of the Italian version of the ABQ.

**Table 3:** Standardized estimates and internal consistency of the Italian translated Athlete Burnout Questionnaire.

Factor	Item	Factor loading	Residual variance	*R* ^2^	α	ω	AVE
Physical/emotional exhaustion	2	0.46	0.79	0.21	0.88	0.81	0.57
	4	0.67	0.55	0.45			
	8	0.87	0.24	0.76			
	10	0.83	0.31	0.69			
	12	0.85	0.27	0.73			
Reduced sense of accomplishment	1	0.35	0.88	0.12	0.81	0.75	0.46
	5	0.83	0.32	0.68			
	7	0.82	0.33	0.67			
	13	0.79	0.38	0.62			
	14	0.46	0.79	0.21			
Sport devaluation	3	0.74	0.45	0.55	0.87	0.85	0.61
	6	0.86	0.26	0.74			
	9	0.94	0.12	0.88			
	11	0.65	0.57	0.43			
	15	0.69	0.52	0.48			

AVE, average variance extracted.

### 3.3 Internal consistency

Reliability estimates for the three ABQ subscales were good (see Table 3). Ordinal Cronbach’s alpha coefficients ranged from 0.81 to 0.88, while McDonald’s omega values ranged from 0.75 to 0.85. Average variance extracted (AVE) ranged from 0.46 to 0.61. Corrected item-total correlations were acceptable for all items (r = 0.48 to 0.82), and average inter-item correlations were all above 0.4, which is considered an adequate value (Clark and Watson, 2016).

### 3.4 Construct validity

As expected, all three burnout dimensions were positively and moderately correlated with perceived general distress levels, and they were negatively correlated or uncorrelated with resilience scores, supporting theoretical expectations (see Table 4).

**Table 4:** Spearman’s correlation coefficients among athlete burnout dimensions and relevant psychological variables.

Variables	General distress	Resilience
Physical/emotional exhaustion	0.38***	−0.01
Reduced sense of accomplishment	0.28***	−0.35***
Sport devaluation	0.26***	−0.08

***p < 0.001.

## 4 Discussion

The present study aimed to validate the Italian version of the ABQ by examining its factorial structure, internal consistency, and construct validity. Through confirmatory factor analyses (CFAs), different competing models were tested: a unidimensional structure, the original three-factor model proposed by Raedeke and Smith (2001), a modified version of the three-factor structure, and a bifactor model. Overall, this study provides preliminary support for the use of an Italian version of the ABQ among Italian athletes. Specifically, a slightly modified three-factor model showed acceptable model fit and outperformed alternative models while preserving the original three-factor structure proposed by Raedeke and Smith (2001).

The unidimensional model, in which all items were constrained to load onto a single latent factor, yielded poor model fit across all indices. This is consistent with theoretical conceptualizations of burnout as a multidimensional construct composed of emotional and physical exhaustion, a reduced sense of accomplishment, and sport devaluation (Raedeke and Smith, 2001). The inadequacy of the unidimensional model confirms that these dimensions should not be collapsed into a single score, as doing so would obscure important differences in athletes’ burnout experiences.

The original three-factor model demonstrated substantially improved model fit, albeit some fit indices were still below conventional thresholds. Inspection of modification indices and standardized residuals indicated localized residual covariances, specifically involving item pairs within the PEE and RSA dimensions. Indeed, items 2 and 4 both address feelings of tiredness related to training or sport participation, whereas items 1 and 14 similarly reflect on achievement and success in sports. The observed residual correlations suggest the presence of shared variance not fully explained by the respective latent factors, likely reflecting a thematic overlap. Allowing these residuals to correlate was therefore both theoretically and empirically justified. At the same time, alternative interpretations should be considered. In particular, these residual correlations may reflect translation-related semantic similarities or potential weaknesses in item formulation. Such patterns can emerge in cross-cultural adaptations independently of translation quality and may signal partial item redundancy. Revising or removing such items could be considered to improve scale clarity or parsimony for future scale refinements, for instance, in the development of a short-form version. However, recent research on the ABQ has shown that most items meaningfully contribute to their respective dimensions and function as core indicators of athlete burnout (Grugan et al., 2024). Thus, removing items solely on the basis on statistical diagnostics may compromise the content validity and theoretical richness of the scale by eliminating subtle yet important facets of the construct.

Incorporating these data-informed and theoretically justified adjustments led to a modified three-factor model that yielded markedly better fit indices, indicating overall acceptable – though not optimal – model fit, as the RMSEA remained slightly above the conventional cutoff. Importantly, all factor loadings were statistically significant and within a theoretically expected range, further supporting the appropriateness of this model. Although some items showed lower loadings, they were retained to preserve content coverage and maintain consistency with the original ABQ structure (Grugan et al., 2024).

Previous validation studies have examined the suitability of bifactor models for the ABQ. For instance, Liu et al. (2022) found that a bifactor model better represented the ABQ factor structure in a sample of Chinese elite athletes (i.e., full-time athletes competing at national or international levels), whereas the original three-factor model was more appropriate for collegiate athletes (i.e., athletes engaged in competitions organized by the university form provincial to national levels). This illustrates how the utility and interpretability of bifactor models may vary depending on the characteristics of the sample and context of use. In our study, the bifactor model - comprising one general burnout factor and three orthogonal specific factors - demonstrated the best absolute fit indices among the tested models. At first glance, this may suggest the presence of an underlying general factor of athlete burnout. However, a closer examination of factor loadings raised some concerns. Notably, several items (e.g., items 3 and 15) exhibited very low or even negative loadings on either the general factor or their respective specific dimension. This undermines the interpretability of the bifactor model, suggesting that, despite its statistical fit, it may not provide a theoretically coherent representation of the construct in our sample. Indeed, it should be considered that traditional fit indices may be biased in favor of bifactor models, as these models provide the most general structure allowing each item to load on both a general and a specific factor (Morgan et al., 2015; Reise, 2012).

Taken together, our findings suggest that the bifactor model provides an empirically competitive representation of the data; however, in the present sample, the bifactor solution showed limited theoretical interpretability. The inconsistent and ambiguous loadings indicate that the bifactor model may be overfitting or failing to capture the intended multidimensional structure of athlete burnout as conceptualized by Raedeke and Smith (2001). For these reasons, the decision to retain the modified three-factor model reflects a theoretically driven choice rather than a purely empirical one. While the existence of a general burnout factor cannot be ruled out on the basis of the present cross-sectional data alone, the three-factor solution provided a clearer and more coherent representation of the construct in this sample, consistent with previous evidence across various cultural contexts, validating the ABQ’s three-factor structure (e.g., Gerber et al., 2018; Grugan et al., 2024; Isoard-Gautheur et al., 2010; Liu et al., 2022). Furthermore, the observed inter-factor correlations – particularly the strong associations involving DEV and the more modest correlation between RSA and PEE – are consistent with the view that burnout dimensions are related yet distinct, each uniquely contributing to the athlete’s experience.

The internal consistency of the three ABQ subscales was satisfactory, indicating good levels of reliability. These results are comparable to previous research that consistently found similar Cronbach’s alpha and McDonald’s omega values (e.g., Cresswell and Eklund, 2006; DeFreese and Smith, 2013; Liu et al., 2022; Raedeke and Smith, 2001). Specific to the Italian context, Vitali et al. (2015) and Morano et al. (2022) reported comparable reliability levels in samples of adolescent athletes. In addition, all items contributed meaningfully to their corresponding latent construct. While the AVE for one of the factors was a bit low, this is not unusual in multidimensional scales with a small number of items per factor. In such contexts, adequate reliability indices and consistent factor loadings may still support acceptable construct representation despite lower AVE values (Hair et al., 2022). Moreover, AVE values were similar to those found by Liu et al. (2022). Nevertheless, a lower AVE may indicate that a substantial proportion of item variance is not captured by the latent factor, suggesting a broader and less homogeneous construct representation, with potential implications for discriminant validity. Although inter-factor correlations were moderate and theoretically consistent, this finding suggests that distinctions between burnout dimensions should be interpreted with appropriate caution. Collectively, these results support that the Italian ABQ possesses acceptable reliability, supporting its use in both research and applied sport psychology contexts.

Construct validity was supported through correlational analyses with relevant external variables. As hypothesized, all three burnout dimensions were positively and moderately associated with psychological distress. This aligns with previous research demonstrating that burnout is closely tied to chronic stress, negative emotional states, and poor mental health outcomes (DeFreese and Smith, 2013; Gerber et al., 2018). Conversely, burnout dimensions were negatively or not significantly associated with resilience, consistent with theoretical expectations. Resilience, typically defined as the ability to adapt and recover from adversity, has been identified as a protective factor against burnout (Fletcher and Sarkar, 2012; Sorkkila et al., 2019).

The current study offers the first psychometric validation of an Italian version of the ABQ, thus filling a significant gap in the literature. Two previous studies used Italian translations of the ABQ among adolescent athletes (Morano et al., 2022; Vitali et al., 2015), however, a full psychometric validation of the instrument has not yet been conducted. Vitali et al. (2015) reported only internal consistency indices, while Morano et al. (2022) conducted a CFA as part of their preliminary analyses but did not compare alternative structural models or provide a detailed evaluation of model fit and item functioning. In contrast, the present study offers the first comprehensive psychometric validation of the ABQ in the Italian context for adult athletes, including a comparison of multiple factor structures (unidimensional, three-factor, bifactor), assessment of model modification, and reliability testing using both Cronbach’s alpha and McDonald’s omega. This more rigorous approach provides stronger empirical support for the use of the ABQ with Italian athletes and ensures the instrument’s conceptual and statistical alignment with the original version proposed by Raedeke and Smith (2001). By confirming its reliability and validity, the present study offers researchers and practitioners a culturally and linguistically appropriate tool to assess burnout among athletes. The findings also underscore the importance of rigorous model testing in cross-cultural adaptations. While the original three-factor structure was broadly supported, the improved fit obtained through data- and theory-based modifications may indicate that established models require careful evaluation, especially in adaptation contexts. To maintain construct integrity and empirical rigor, statistical modeling should also take into account theoretical factors such as overlap in item content. At the same time, such model modifications may reflect sample- or translation-specific characteristics and should therefore be interpreted with caution. Accordingly, the present modified three-factor solution should be viewed as provisional and preliminary, requiring replication and cross-validation in different samples before being considered a stable representation of the ABQ structure in the Italian context.

Despite its strengths, the study is not without limitations. A critical limitation of the present study concerns the lack of measurement invariance testing across relevant subgroups. Although the sample size was adequate for CFA, it was relatively modest for testing more complex models, such as multi-group CFA, particularly given the uneven distribution of participants across gender, sport type, and competitive level. The sample was predominantly composed of male athletes, individuals engaged in team sports, and athletes competing at higher competitive levels, which precluded the examination of configural, metric, and scalar invariance across these subgroups. This imbalance across subgroups may also limit the representativeness and generalizability of the findings. In addition, preliminary subgroup comparisons revealed small and dimension-specific differences in observed ABQ scores. While these findings provide initial information regarding potential sources of variability, they should be interpreted with caution, as the equivalence of the measurement properties across subgroups cannot be formally assumed. Consequently, cross-group comparisons of burnout scores based on the Italian ABQ should be considered tentative. Future research should, therefore, examine the psychometric properties of the Italian ABQ on larger and more diverse samples and test measurement invariance across subgroups such as gender, sport type (individual vs. team), competitive level (i.e., professional vs. semi-professional athletes), and age (youth vs. adults). Such efforts are necessary to establish the broader generalizability and external validity of the instrument, particularly for female athletes, athletes involved in individual sports, youth athletes, and those competing at recreational or lower competitive levels. Second, our analyses primarily relied on CFA models, which impose strict assumptions such as zero cross-loadings. Given the strong relationships between some ABQ dimensions, cross-loadings may exist, causing CFA to oversimplify the burnout structure and fit the data poorly. Researchers should consider implementing complementary statistical approaches in their studies, like item response theory (IRT) or ESEM, that could offer deeper insights into item functioning and factor structure. Third, while resilience and distress are relevant constructs in the burnout context, expanding the nomological network - including constructs such as motivation, competitive anxiety, and athlete engagement - would further strengthen the convergent and discriminant validity of the questionnaire. Longitudinal studies are also needed to establish the predictive validity and test–retest reliability of the Italian ABQ, which were not addressed in the present study. In addition, all variables were assessed via self-report measures, which may have introduced social desirability bias. Finally, while the translation followed standard procedures, no formal cognitive interviewing or pilot testing with athletes was conducted prior to data collection, hence limiting conclusions regarding item clarity and cultural adaptability.

Therefore, the present validation should be regarded as preliminary, as it focuses on the internal structure of the Italian ABQ while not addressing measurement invariance across groups, temporal stability, or longitudinal and predictive validity; consequently, the available evidence does not yet allow to determine whether the instrument operates equivalently across different groups or to use it with confidence for monitoring changes in burnout over time.

## 5 Conclusion

The present study represents a critical step toward establishing a valid and reliable Italian version of the Athlete Burnout Questionnaire. The findings support the use of the ABQ as a multidimensional measure of athlete burnout in Italian sport contexts. The instrument demonstrates sound psychometric properties, including a stable three-factor structure, good reliability, and expected associations with psychological constructs. The validated scale offers researchers and practitioners a robust tool for assessing burnout in Italian-speaking adult athletes and facilitates cross-cultural comparisons in sport psychology research. These preliminary results encourage further research to confirm and extend the applicability of the ABQ across broader Italian athletic populations and longitudinal settings.

## Data Availability

The raw data supporting the conclusions of this article will be made available by the authors, without undue reservation.

## Appendix A

### The Italian ABQ

Per favore, leggi attentamente ogni affermazione e decidi se ti senti mai in questo modo riguardo alla tua attuale pratica sportiva. La tua attuale pratica sportiva include tutti gli allenamenti e le competizioni che hai svolto durante questa stagione. Indica con quale frequenza hai provato questa sensazione o hai avuto questo pensiero durante la stagione scegliendo un numero da 1 a 5, dove 1 significa “Non mi sento quasi mai in questo modo” e 5 significa “Mi sento in questo modo quasi sempre”. Non ci sono risposte giuste o sbagliate, quindi rispondi a ogni domanda nel modo più onesto possibile. Per favore, assicurati di rispondere a tutte le domande.

**Table:**

	*Quanto spesso ti senti così?*	Quasi mai	Raramente	Qualche volta	Spesso	Quasi sempre
1	Sto realizzando molte cose di valore nello sport	1	2	3	4	5
2	Mi sento così stanco/a dal mio allenamento che faccio fatica a trovare l’energia per fare altre cose	1	2	3	4	5
3	L’impegno che spendo nello sport sarebbe meglio speso facendo altre cose	1	2	3	4	5
4	Mi sento eccessivamente stanco/a per via della mia partecipazione sportiva	1	2	3	4	5
5	Non sto ottenendo molto nello sport	1	2	3	4	5
6	Non mi importa più della mia performance sportiva come una volta	1	2	3	4	5
7	Non sto rendendo all’altezza della mia capacità nello sport	1	2	3	4	5
8	Mi sento sfinito/a dallo sport	1	2	3	4	5
9	Non sono preso/a dallo sport come una volta	1	2	3	4	5
10	Mi sento fisicamente consumato/a dallo sport	1	2	3	4	5
11	Mi sento meno preoccupato/a di avere successo nello sport rispetto a una volta	1	2	3	4	5
12	Sono esausto/a dalle richieste mentali e fisiche dello sport	1	2	3	4	5
13	Sembra che nonostante quello che faccia, io non renda bene come dovrei	1	2	3	4	5
14	Mi sento di avere successo nello sport	1	2	3	4	5
15	Provo sentimenti negativi verso lo sport	1	2	3	4	5
